# Incidence of Type 2 Diabetes in Kuwaiti Children and Adolescents: Results From the Childhood-Onset Diabetes Electronic Registry (CODeR)

**DOI:** 10.3389/fendo.2019.00836

**Published:** 2019-12-04

**Authors:** Hessa Al-Kandari, Dalia Al-Abdulrazzaq, Lena Davidsson, Prem Sharma, Abeer Al-Tararwa, Fawziya Mandani, Faisal Al-Shawaf, Fatma Al-Hussaini, Mariam Qabazard, Dania Haddad, Maria Al-Mahdi, Fahad Al-Jasser, Ayed Alanezi, Hala Al-Sanea, Iman Al-Basari, Afaf Al-Adsani, Azza Shaltout, Mejedah AbdulRasoul

**Affiliations:** ^1^Department of Population Health, Dasman Diabetes Institute, Kuwait City, Kuwait; ^2^Department of Pediatrics, Farwaniya Hospital, Ministry of Health, Sabah Al Nasser, Kuwait; ^3^Department of Pediatrics, Faculty of Medicine, Kuwait University, Kuwait City, Kuwait; ^4^Department of Pediatrics, Mubarak Al-Kabeer Hospital, Ministry of Health, Jabriya, Kuwait; ^5^Special Services Facilities, Dasman Diabetes Institute, Kuwait City, Kuwait; ^6^Department of Pediatrics, Sabah Hospital, Ministry of Health, Kuwait City, Kuwait; ^7^Department of Pediatrics, Adan Hospital, Ministry of Health, Hadiya, Kuwait; ^8^Department of Pediatrics, Amiri Hospital, Ministry of Health, Kuwait City, Kuwait; ^9^Department of Pediatrics, Jahra Hospital, Ministry of Health, Al Jahra, Kuwait; ^10^Department of Medicine, Sabah Hospital, Ministry of Health, Kuwait City, Kuwait

**Keywords:** diabetes, type 2 diabetes, childhood, childhood type 2 diabetes, Kuwait

## Abstract

**Background:** Type 2 Diabetes (T2D) in children and adolescents has become an important public health concern due to the increase in childhood obesity worldwide. The urgency to address T2D is evident as children and adolescents are at a higher risk of complications due to prolonged disease duration. We aimed to estimate the incidence rate (IR) of T2D in Kuwaiti children and adolescents aged 14 years and younger between 2011 and 2013 and to describe their clinical characteristics at the time of diagnosis.

**Material and Methods:** All newly diagnosed patients were registered through the Childhood-Onset Diabetes electronic Registry implemented in Kuwait. Cases who met the 2018 ISPAD guidelines for diagnosis of T2D were included.

**Results:** A total of 32 patients were included, equally distributed gender-wise, with a mean age 12.2 years (±1.7 SD), lower for females than males (11.5 vs. 12.2, *p* < 0.025). Data ascertainment was 94.1% (95%CI; 91.6–96.6%). Overall IR was 2.56 (95% CI; 1.78-3.56) per 100,000 Kuwaiti children and adolescents per year. Most of the patients (*n* = 30; 93.8%) presented with T2D between the ages 10–14 years, with age-specific IR of 8.0 (95%CI; 5.5–11.3). No statistically significant difference between males and females with regards to BMI z scores or HbA1C at diagnosis.

**Conclusion:** The true incidence of T2D in Kuwaiti children and adolescents is expected to be considerably higher as we have reported only symptomatic cases. Future research should focus on screening children and adolescents at risk to enable accurate estimates. More efforts are needed to better understand the clinical course of T2D early in life to improve management, prevent complications and improve quality of life.

## Background

Type 2 Diabetes (T2D) in children and adolescents has become an increasingly important public health concern throughout the world (1–10) due to the recent alarming increase in childhood obesity worldwide (11). In a recent study, 30.5% of children and adolescents aged 6–18 years in Kuwait were found to be obese (12). However, only sparse population-based data on T2D in children and adolescents have been reported leading to unique challenges in assessing the extent of the problem, developing relevant health strategies, and evaluating the quality of care provided for these patients. In particular, the lack of evidence-based diagnostic criteria for T2D in this age group represent a major challenge. It is notable that available pediatric guidelines have not been validated in children and adolescents but are based on extrapolations from adult cut-off values for biomarkers (1).

The urgency to address T2D early in life is evident as children and adolescents are at a higher risk of complications compared to adults due to prolonged disease duration. This in turn leads to a significant burden on—not only the patient—but also the family, the community, and the national healthcare system (13, 14). Therefore, providing accurate data on the incidence and prevalence of T2D in children and adolescents is a crucial starting point in tackling this global pandemic. The aim of this study was to estimate the incidence rate (IR) of T2D in Kuwaiti children and adolescents aged 14 years and younger between 2011 and 2013 and to describe their clinical characteristics at the time of diagnosis. The data are based on the Childhood-Onset Diabetes electronic Registry (CODeR) implemented in Kuwait.

## Materials and Methods

Kuwait is a small country overlooking the Arabian Gulf and is home to a total population of 3,588,092 of whom 1,177,071 (32.8%) were Kuwaitis as per the Public Authority of Civil Information (PACI), 2013. CODeR is a comprehensive prospective population-based diabetes registry system maintained by Dasman Diabetes Institute in collaboration with the Ministry of Health (MOH), established in 2011 for surveillance of children and adolescents with diabetes diagnosed in the country. Details of the implementation of the registry are described by Shaltout et al. (15).

All newly diagnosed cases are registered electronically or manually in the registry from all over the state of Kuwait. Patients are reported by physicians from a primary source (local governmental and private hospitals) and a secondary source (the Kuwait Diabetes Society, MOH primary care centers, and Dasman Diabetes Institute). Data were extracted using standard registry data forms and the medical records of patients registered as T2D were reviewed. The data included baseline information at the time of diagnosis such as demographic information, anthropometric measures (weight, height, and body mass index [BMI]) expressed as standard deviation scores (SDS) according to the World Health Organization (WHO) child growth standards (16), as well as measures of Hemoglobin A1C (HbA1C), fasting or random blood sugar, C-peptide, insulin, pancreatic autoimmune antibodies. The registry also captures information on family history of T2D.

**Figure 1:**
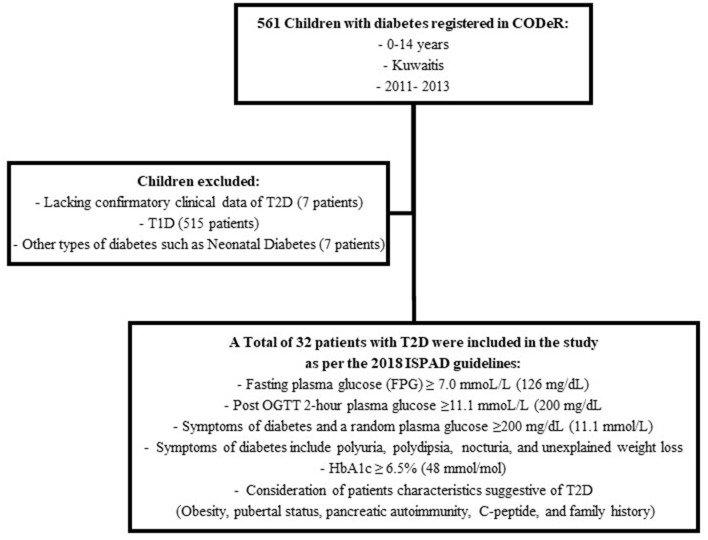
Diagnosis of T2D in children and adolescents registered in CODeR.

The diagnosis of T2D was reviewed by two board certified pediatric endocrinologists as per the 2018 International Society of Pediatric and Adolescent diabetes (ISPAD) guidelines (Figure 1) (1).

Information on the total number of Kuwaiti children and adolescents in different age groups was obtained from the official statistics provided by PACI for 2011 to 2013. The ascertainment with 95% confidence interval (CI) was calculated using Chapman's formula (17, 18), $n=\frac{\left(L1+1\right)\;\left(L2+1\right)}{\left(d+1\right)}-1$, for two-list capture-recapture method. Here, list L1 is based on number of patients from primary source (governmental and private hospitals), and list L2, number of patients based on secondary source (Kuwait Diabetes Society, MOH primary care centers, and Dasman Diabetes Institute), while d, the duplicates in the lists. The study was approved by the Ethics Review Committee at Dasman Diabetes Institute in Kuwait.

Statistical analysis was carried out using the computer software “Statistical Package for Social Sciences, SPSS version 25.0” (IBM Corp, Armonk, NY, USA). The quantitative or continuous variables were first ascertained for normal distribution assumption, applying the Kolmogorov-Smirnov test, and have been presented as mean ± standard deviation (SD). Gender-wise comparisons were made using independent *t*-test. The overall or age-specific as well as gender-wise, IR for T2D in Kuwaiti children and adolescents were calculated as cases per 100,000 Kuwaiti children and adolescents per year and presented with 95% confidence interval (CI). The two-tailed probability value “*p*” < 0.05 was considered statistically significant.

## Results

A total of 39 patients were registered as T2D during the study period. However, seven patients were excluded due to lack of confirmatory data (Figure 1). Thus, 32 patients met the criteria for diagnosis of T2D as per the 2018 ISPAD guidelines. The ascertainment rate was 94.1% (95%CI; 91.6–96.6%). The overall incidence rate for ages 0–14 years was 2.56 (95%CI; 1.78–3.56) per 100,000 Kuwaiti children and adolescents per year (Table 1). The incidence rates with 95% CI, for the years 2011, 2012, and 2013 were found to be 3.42 (1.95–5.61), 2.64 (1.39–4.58), and 1.65 (0.78–3.56) respectively. The mean annual age- and gender-specific incidence rates per 100,000 Kuwaiti children and adolescents per year of T2D in Kuwaiti children and adolescents during the study period is presented in Table 1. Most of the patients (*n* = 30; 93.8%) presented with T2D between the ages of 10–14 years, with age-specific incidence rate of 8.0 (95%CI; 5.5–11.3) per 100,000 Kuwaiti children and adolescents per year. Only two patients were below the age of 10 years (6.5 and 8.0 years, respectively) at the time of diagnosis.

**Table 1:** Mean annual age- and gender-specific incidence rates per 100, 1,000 Kuwait children and adolescents per year of T2D between 2011 and 2013.

	**Population**	**Cases**	**IR**	**95% CI**
**Males**				
5–10 years	210,728	0	0	—
10–14 years	191,942	16	8.34	(4.93–13.25)
0–14 years	639,125	16	2.50	(1.48–3.98)
**Females**				
5–10 years	202,175	2	0.99	(0.17–3.27)
10–14 years	183,314	14	7.64	(4.35–12.51)
0–14 years	612,309	16	2.61	(1.55–4.15)
**Total**				
5–10 years	412,903	2	0.48	(0.08–1.60)
10–14 years	375,256	30	8.00	(5.49–11.27)
0–14 years	1,252,434	32	2.56	(1.78–3.56)

*T2D, Type 2 diabetes; IR, Incidence rate; 95% CI, 95% Confidence interval*.

The male to female ratio was 1.0, i.e., 16 cases were male and 16 females. Baseline characteristics of patients with T2D are summarized in Table 2. At diagnosis, the mean age was 12.2 ± 1.7 years, somewhat lower in females, 11.5 ± 2.0 years as compared to male patients 12.9 ± 1.2 years (*p* = 0.025). The youngest patient registered was a female aged 6 years and 6 months (BMI 36.1 kg/m^2^ (+5.16 SDS), HbA1C 8.9%, 1461 pmol/L (Reference: 160–1,100), 27.99 μIU/ml (Reference: 1.9–23), positive anti-GAD and positive anti-Insulin antibodies). There was no statistically significant difference between males and females with regards to BMI or HbA1C at diagnosis (Table 2).

**Table 2:** Baseline characteristics of Kuwaiti children and adolescents diagnosed with T2D between 2011 and 2013.

**Variable**	**Total (*N* = 32)**	**Males (*n* = 16)**	**Females (*n* = 16)**	***P*-value**
**Age at diagnosis**				
Years, mean (SD)	12.2 (1.7)	12.9 (1.2)	11.5 (2.0)	0.025
BMI, SDS (SD)^*^	+3.07 (0.7)	+3.09 (0.6)	+3.05 (0.8)	0.873
HbA1C, % (SD)^♦^	9.9 (2.7)	10.0 (2.8)	9.7 (2.7)	0.774

T2D, Type 2 diabetes; BMI, Body mass index; HbA1C, hemoglobin A1C.

* Missing for 2 cases (2 Males).

♦ *Missing for 1 case (1 Male)*.

## Discussion

This study aimed at estimating the incidence of T2D in Kuwaiti children and adolescents between 2011 and 2013 and describing their baseline characteristics at the time of diagnosis. The overall incidence rate during the study duration was 2.56 per 100,000 Kuwait children and adolescents per year. Unfortunately, no previously reported data are available for comparison in Kuwait. In Qatar, a neighboring country with similar culture and lifestyle, an incidence rate of 2.72 per 100,000 was reported in 2016 for children and adolescents under the age of 14 years (7). Elsewhere in the world, for example in China, Canada, and New Zealand, reports of lower incidence rates have been published; 1.96 per 100,000 in 5–19 years old (19), 1.54 per 100,000 in < 18 years (20), and 1.3 per 100,000 in < 15 years (21), respectively. In 2014, Denmark reported the diagnosis of T2D in 7 young individuals and an overall prevalence of 0.6 per 100,000 (22). However, the US has reported a higher incidence, 12.5 per 100,000 in 10-19 year old children and adolescents, based on the SEARCH for Diabetes in Youth Study between 2011 and 2012 (5). Very few studies report on incidence of T2D in different ethnic groups although data from the UK indicate the importance of racial and ethnic differences as the incidence of T2D in black children and adolescents in England was higher (3.9 per 100,000) compared to whites (0.35 per 100,000) based on data 2004-5 (23).

It should be noted that the incidence reported in our study is only representing symptomatic cases, i.e., those presenting with symptoms of diabetes like polyuria and polydipsia to health-care centers, and thus does not reflect the true incidence rate of T2D in children and adolescents as asymptomatic manifestation of T2D is common in childhood and can be diagnosed by screening programs (11).

The mean age of diagnosis of T2D during the study period was 12.2 years (± 1.7SD). Our data are consistent with international reports of youth-onset T2D which typically occurs during the second decade of life with a median onset at 13.5 years (1). This phenomenon is explained by the physiologic peak of insulin resistance that occurs during puberty in which insulin sensitivity decreases by around 30% (24, 25). Therefore, girls usually present with T2D earlier than boys, which is consistent with our findings (Table 2), (26, 27). T2D rarely occurs in pre-pubertal children as found in our study where only two patients (6.3%) presented below the age of 10 years (6.5 and 8.0 years, respectively) (26, 27). Nevertheless, recent international reports suggest that the age of onset of T2D in children and adolescents occurs at an increasingly younger age. For example, in Qatar, 24.1% of children and adolescents with T2D diagnosed during 2012-2016 were between the ages of 5 and 10 years (7) and a more rapid increase of the annual incidence rate of T2D has been reported for children and adolescents aged 0–14 years (37.4%) as compared with 15–19 year olds (18.1%) between 1991 and 2006 in the UK (28). Similarly, three out of the seven Danish children reported with T2D were diagnosed in the pre-pubertal period (22).

In our data set, the youngest patient with T2D was obese with positive pancreatic autoimmunity. Fifteen to 40% of patients with T2D have pancreatic autoimmunity and are referred to as autoimmune T2D (1, 29–31). However, children and adolescents with autoimmune T2D are reported to be less overweight and are less likely to be female, thus our patient is not representative of autoimmune T2D (31). Patients such as this young girl represent a diagnostic challenge as it can be argued that she might have autoimmune type 1 diabetes that presented with underlying insulin resistance due to obesity.

This is the first report on the incidence of T2D in children and adolescents in Kuwait. We recognize the limitations to our study as the duration of the registry was relatively short. Clearly, it would be important to monitor the incidence trends of T2D over a longer period, especially in countries with high prevalence of childhood obesity to assess the magnitude of the problem. Furthermore, due to the lack of genetic testing in the registry, patients with possible diagnosis of Maturity-Onset Diabetes of the Young (MODY) could not be accounted for and might be mis-diagnosed as T2D.

In conclusion, we have reported the incidence of T2D as 2.56 per 100,000 Kuwaiti children and adolescents per year between 2011 and 2013. The true incidence of T2D in Kuwaiti children and adolescents is expected to be considerably higher as we have reported only on symptomatic cases. Increased awareness of T2D in children and adolescents is needed among healthcare providers, in particular when caring for obese children and adolescents. Future research should focus on screening children and adolescents at risk of T2D to enable accurate estimates of incidence and prevalence. Furthermore, more efforts are needed to better understand the clinical course of T2D early in life to improve management and prevent complications so that children and adolescents living with T2D from an early age will be able to enjoy good quality of life.

## Data Availability Statement

The datasets for this manuscript are not publicly available because the data was obtained from the Childhood-onset Diabetes electronic Registry (CODeR) owned by Dasman Diabetes Institute and sharing the dataset was not approved by the Ethics Review Committee at the institute. Requests to access the datasets should be directed to HA-K (email: hessa.alkandari@dasmaninstitute.org).

## Ethics Statement

The studies involving human participants were reviewed and approved by Ethics Review Committee at Dasman Diabetes Institute. Written informed consent to participate in this study was provided by the participants' legal guardian/next of kin.

## Author Contributions

HA-K and DA-A made the main contribution to the conception and design, acquisition of data, analysis and interpretation of data, and drafted the manuscript. LD contributed to the drafting of this manuscript. PS contributed to the analysis of the data and drafting of the manuscript. AA-T, FM, FA-S, FA-H, MQ, DH, MA-M, FA-J, AA, HA-S, IA-B, AA-A, and MA contributed to the acquisition of data and reviewed the manuscript. AS contributed to the conception and design and reviewed the manuscript.

### Conflict of Interest

The authors declare that the research was conducted in the absence of any commercial or financial relationships that could be construed as a potential conflict of interest.

## Abbreviations

CODer: Childhood-Onset Diabetes electronic Registry
T2D: Type 2 diabetes
T1D: Type 1 Diabetes
ISPAD: International Society of Pediatric and Adolescent diabetes
OGTT: Oral glucose tolerance test
HbA1C: Hemoglobin A1C.
